# Cladodes: Chemical and structural properties, biological activity, and polyphenols profile

**DOI:** 10.1002/fsn3.2388

**Published:** 2021-06-10

**Authors:** Madeleine Perucini‐Avendaño, Mayra Nicolás‐García, Cristian Jiménez‐Martínez, María de Jesús Perea‐Flores, Mayra Beatriz Gómez‐Patiño, Daniel Arrieta‐Báez, Gloria Dávila‐Ortiz

**Affiliations:** ^1^ Departamento de Ingeniería Bioquímica Escuela Nacional de Ciencias Biológicas Instituto Politécnico Nacional (IPN) Unidad Profesional Adolfo López Mateos Zacatenco Delegación Gustavo A. Madero Ciudad de México México; ^2^ Centro de Nanociencias y Micro y Nanotecnologías Instituto Politécnico Nacional (IPN) Unidad Profesional Adolfo López Mateos Zacatenco Delegación Gustavo A. Madero Ciudad de México México

**Keywords:** analytical methods, cladodes, health effects, hierarchical structure, *Opuntia ficus‐indica*, polyphenols

## Abstract

The nopal cactus is an essential part of the Mexican diet and culture. The per capita consumption of young cladodes averages annually to 6.4 kg across the nation. In addition to contributing to the country's food culture, the nopal is considered a food with functional characteristics since, in addition to providing fiber, an important group of polyphenolic compounds is present, which has given cladodes to be considered a healthy food, for what they have been incorporated into the diet of Mexican people and many other countries worldwide. Research suggests that polyphenols from cladodes act as antioxidants and antidiabetics. This review studies the main phenolic components in cladodes and summarizes both conventional and novel methods to identify them.

## Graphical abstract

This manuscript summarizes the different polyphenols that have been elucidated in the cladodes to which various biological activities with benefits to human health are attributed, as well as the main factors that affect the processes of preparation and extraction.

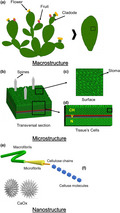

## 1 INTRODUCTION

Worldwide, nopal has become a valuable crop due to its health benefits, ease of cultivation, marketing, and climate adaptation (Aruwa et al., 2018). Nopal (*Opuntia ficus‐indica* (L.) Mill) belongs to the Cactaceae family that comprises about 1,500 species (El‐Mostafa et al., 2014), some of these species are *Opuntia: basilaris, chlorotica, engelmannii, fragilis, humifusa, leucotricha, macrocentra, macrorhiza, dillenii, santa‐rita, stricta* (Majdoub et al., 2001). Its cultivation represents a major source of income for farmers living in semi‐arid regions (Bayar et al., 2016). Nopal can grow in South America and other dry areas such as Africa, Australia, Southern Europe, and Asia (Khouloud et al., 2018; Majdoub et al., 2001). Nevertheless, Mexico accounts for 90% of the world´s production and represent the largest supplier to the United States, Canada, Japan, and European countries. Per capita consumption of nopal in Mexico is 6.4 kg (FAOSTAT, 2016).

Nopal is one of the most consumed species due to its nutritional value (Majdoub et al., 2001); furthermore, recent trends in healthy food consumption aroused scientists’ interest to study the effects of nopal polyphenolic compounds in oxidative stress‐related diseases (Scalbert et al., 2005). This work describes the nopal as a potential source of polyphenols and the main factors affecting their analytical identification. In addition, we highlight the importance of the relationship structure function in promoting health through cladodes consumption.

## 2 NOPAL: MORPHOLOGICAL DESCRIPTION

**FIGURE 1:**
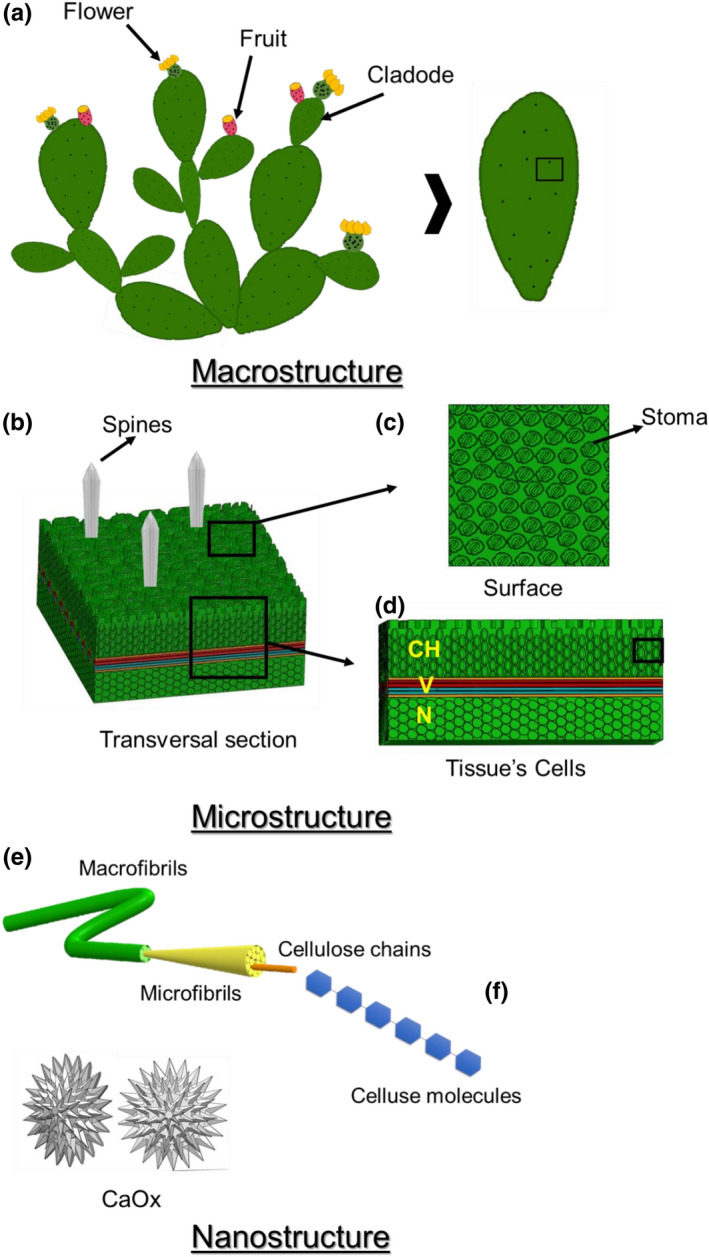
Hierarchical structural organization of nopal

The hierarchical structural organization evaluates the structure functioning at different dimensional scales (macrostructure, microstructure, and nanostructure) (Gibson, 2012). Figure 1 shows a model of the hierarchical structural organization of the nopal, which contributes to its morphological description.

At the first level (macrostructure), nopal has three components which are as follows: flowers, prickly pears fruits, and leaves (botanically called cladodes) (Salehi et al., 2019). The flowers are pear‐shaped, which allows insect pollination (Small & Catling, 2004). The prickly pears are usually ovoid and spherical, often green, yellow, or bright red. They have a high number of seeds, and a protective shell covered with small spines. This gives them an important role in the genetic diversity and distribution of the species (Carrillo et al., 2017).

The leaves or cladodes (Figure 1a) are ovoid or elongated racquet‐shaped, with 30–60 cm in length depending on the water and nutrients available (Ciriminna et al., 2019). In Africa, cladodes are exclusively used for animal feeding (De Albuquerque et al., 2019; Mounir et al., 2020); while in Japan, they are hydroponically cultivated for human consumption (Horibe, 2018), as a medicinal plant for diabetes and hypercholesterolemia (Santos‐Zea et al., 2011). Cladodes have areolas from where flowers, fruits, and thorns grow. One to five large, hard spines, and multiple smaller ones (glochidia) protect cladodes against light reflection, water loss, and herbivores predation (Marin‐Bustamante et al., 2018).

The epidermis (Figure 1b) contains numerous stomata (Figure 1c) that control photosynthesis and respiration (Salem‐Fnayou et al., 2014). An inner tissue called chlorenchyma (CH) constitutes the second hierarchical level (microstructure), which consists of green plastids and abundant starch. The vascular tissue (V) located at the chlorenchyma tissue and the nucleus tissue (N) junction serves as a water and nutrient transporter into the plant, allowing the tissue to function as water storage for long periods of drought (Ginestra et al., 2009). The colorless central core tissue contains reserves of carbohydrates, proteins, and polyphenols (Feugang et al., 2006).

At the third hierarchical level (nanostructure), the macro cellulose fibers provide structure to the cell wall (Figure 1e). Alongside the tissues, calcium oxalate crystals are found (decreasing in content as cladodes mature) making calcium more bioavailable in younger cladodes (Contreras‐Padilla et al., 2016), which are consumed as vegetables in different stages of maturation ranging from 30 to 90 days (Hernandez‐Becerra et al., 2020; Marin‐Bustamante et al., 2018). Finally, on the fourth hierarchical level, we find the cellulose molecular structure (Figure 1f) (Ventura‐Aguilar et al., 2017).

## 3 CLADODE: COMPOSITION AND BIOLOGICAL ACTIVITY

**FIGURE 2:**
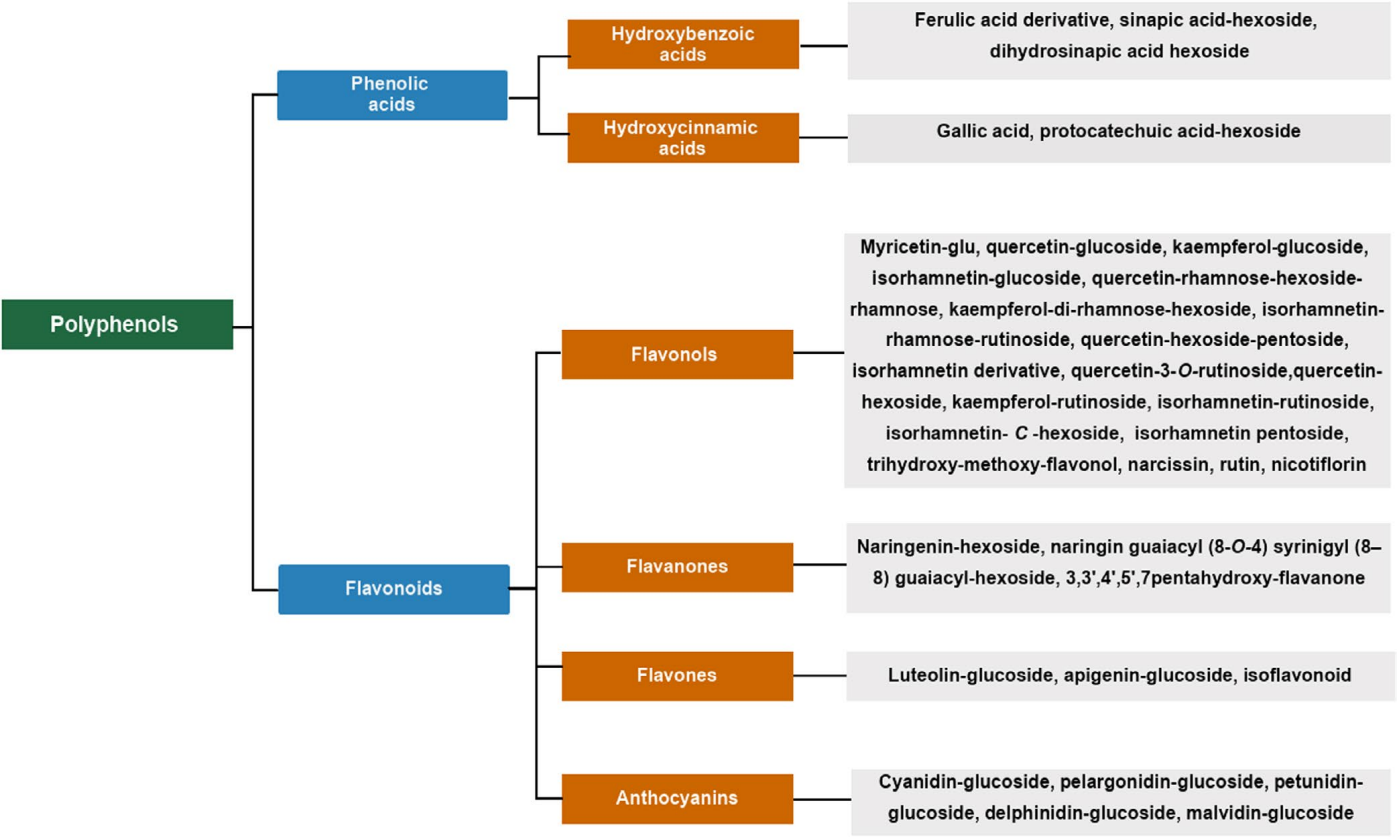
Structural proposal of the Opuntia ficus‐indica mucilage

Cladode chemical composition may vary according to soil factors, cultivation season, and plant age (Table 1). The primary metabolites of cladodes are water, carbohydrates, and proteins. The carbohydrates in cladodes are divided into two types: (a) structural ones that are part of the cell wall, as cellulose (21.6 wt%), hemicelluloses 8.19%, and lignin (3.6 wt%) (López‐Palacios et al., 2016; Scaffaro et al., 2019), and (b) the storage carbohydrates constituted by monosaccharides such as arabinose, galacturonic acid, glucuronic acid, galactose, glucose, xylose, rhamnose, mannose, and fructose (Rodríguez‐González et al., 2014). Polysaccharides from *Opuntia ficus‐indica* *(*L.*)* Mill plants build molecular networks with the capacity to retain water, thus they act as mucoprotective agents (Di Lorenzo et al., 2017). Mucilage is the main polysaccharide of cladodes, it contains polymers of β‐d‐galacturonic acid bound in positions (1–4) and traces of R‐linked l‐rhamnose (1–2) (Figure 2) (Quinzio et al., 2018). Mucilage regulates both the cell water content during prolonged drought and the calcium flux in the plant cells (Hernández‐Urbiola et al., 2010). In the food industry, mucilage is used as an additive, an emulsifier, and an edible coating to extend the shelf life of food products (Medina‐Torres et al., 2013).

**TABLE 1:** Chemical composition of nopal cladodes

Composition (% DW)					Reference
Carbohydrates	Proteins	Lipids	Crude fiber	Ash	
42.94	7.07	2.16	7.07	17.65	Hernandez‐Urbiola et al. (2010)
—	11.73	1.89	55.05	23.05	Cornejo‐Villegas et al. (2010)
61.4	6.7	0.1	15.0	17.3	Guevara‐Fig ueroa et al. (2010)
38.0	11.2	0.69	5.97	14.4	Astello‐García et al. (2015)

Note

—, No determinate; DW, dry weight.

Cladodes contain around 6.7%–11.73% of protein (Table 1). Amino acids such as alanine, isoleucine, and asparagine are found in young cladodes, whereas threonine prevails only in mature cladodes (Figueroa‐Pérez et al., 2018). Young cladodes have a higher protein content than mature cladodes, which may be related to the increased metabolic activity in the early stages of maturation (Nuñez‐López et al., 2013). Furthermore, analyses of plant extracts of the Cactaceae family identified several enzymes (e.g., lipases, proteinases, and glucosidases) (Guevara‐Figueroa et al., 2010), and a large content of minerals (23.05%).

Over the years, Mexican people have developed several chronic degenerative diseases such as obesity, diabetes, and cardiovascular diseases (Aparicio‐Saguilán et al., 2015). Traditional Mexican medicine recommends consuming cladodes due to their bioactive compounds' effects on health (Table 2); for example, the ability of polyphenols to eliminate free radicals (De Santiago et al. 2019; Filannino et al., 2016; Kim et al., 2016; Petruk et al. 2017).

**TABLE 2:** Biological activities reported in cladodes

Biological activity	Bioactive compound	Reference
Anti‐Inflammatory	Isorhamnetin glycosides^a^	Antunes‐Ricardo et al. (2017)
	Isorhamnetin conjugates^a^ Flavonoids	Antunes‐Ricardo et al. (2015) Filannino et al. (2016)
Antidiabetic	Flours obtained from different maturity stages	Nuñez‐López et al. (2013)
	Carbohydrates and leucine^b^	Deldicque et al. (2013)
Antimicrobial and Antibiofilm Activity	Polyphenols^c^	Avila‐Nava et al. (2017)
Antioxidants	Polysaccharides	Nuñez‐López et al. (2013)
	Dehydrated cladode	López‐Romero et al. (2014)
	Polyphenols^d^	Avila‐Nava et al. (2014)
	Flavonoids	Filannino et al. (2016)
	Polyphenols^e^	Msaddak et al. (2017)
	Polyphenols^e^	Smida et al. (2017)
	Polyphenols^f^	Kechebar et al. (2017)
	Polyphenols^c^	Smida et al. (2017)
	Polyphenols^b^	Petruk et al. (2017)
	Polyphenols^c^	Andreu et al. (2018)
	Polyphenols^g^	De Santiago et al. (2018)
	Quercetin, isorhamnetin and kaempferol^e^	Salehi et al. (2019)
	Fermented cactus cladodes	De Santiago et al. (2019)
Hypoglycemic properties	Flours obtained from different maturity stages	Slimen et al. (2019)
Hypercholesterolemia	Isorhamnetin derivatives and piscidic acid^f^	Antunes‐Ricardo et al. (2017)
Neuroprotective activity	Polyphenols^c^	Antunes‐Ricardo et al. (2015)
Immunoprotective	Polyphenols^c^	Nuñez‐López et al. (2013)
Thermoprotective properties	Betanin^b^	Deldicque et al. (2013)
Antiproliferative in human colon carcinoma	Polyphenols^f^	Serra et al. (2013)
Prebiotic potential	Polyphenols, Cladodio	Sánchez Tapia et al. (2017)

Note

Dissolvent used in the extraction: ^a^NaOH, ^b^water, ^c^methanol, ^d^acidified methanol, ^e^methanol:acetone:water, ^f^ethanol, ^g^methanol: acidified water.

Avila‐Nava et al. (2014) assessed the antioxidant capacity of cladodes both in vitro and in vivo, by evaluating the consumption of cladodes for 3 days (300 g/day) in healthy subjects aged 20–30 years, with a body mass index (BMI) <25 kg/m^2^. The results showed an increase in the antioxidant activity of blood (↑5%) and plasma (↑20%). The polyphenols quercetin, isorhamnetin, and kaempferol were identified by high‐performance liquid chromatography (HPLC). The authors concluded that consuming cladodes can reduce pathologies associated with reactive oxygen species.

Additionally, Petruk et al. 2017 found that eucomic and piscidic acids obtained from cladodes polyphenols were responsible for antioxidant activity and produced a protective effect against apoptosis of human keratinocytes induced by UVA. Scholars classified cladodes as a functional food and a prebiotic since they modify the gut microbiota, reduce metabolic endotoxemia, and other obesity and metabolic syndrome biochemical abnormalities (Angulo‐Bejarano et al., 2014; Mercado‐Mercado et al., 2019; Sanchez‐Tapia et al., 2017). Cladodes have antimicrobial, anticancer, and antidiabetes activity and protective effects on hypertension, hypercholesterolemia, rheumatic pain, antiulcerogenic activity, gastric mucosa diseases, and asthma (Tahir et al. 2019). These beneficial health outcomes are attributed to some components of cladodes: polyphenols (phenolic acids, flavonoids, and anthocyanins), β‐carotene, oligosaccharides, polysaccharides, sterols, lignans, saponins, and some vitamins such as E and C (du Toit et al. 2018).

## 4 POLYPHENOLS: EXTRACTION AND IDENTIFICATION METHODS

**FIGURE 3:**
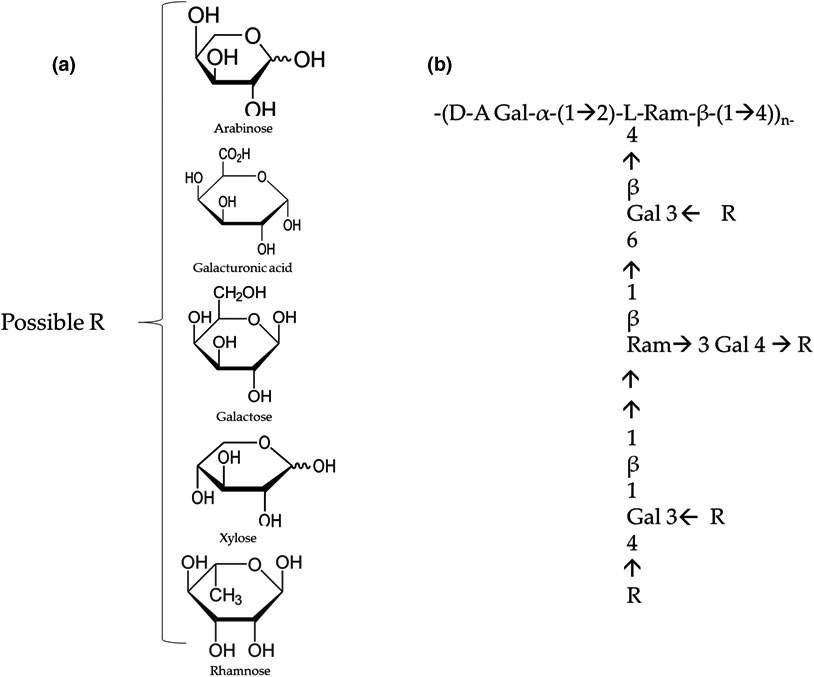
Polyphenols in cladodes

Polyphenols constitute one of the largest groups of secondary plant metabolites (Galanakis et al., 2018; Mirali et al., 2017). They contain one or more hydroxyl groups linked to a benzene ring and have an essential function in the defense against plant pathogens and abiotic stressors (López‐Romero et al., 2014). Figure 3 shows the main polyphenols identified over time in cladodes by different analytical techniques (Antunes‐Ricardo et al., 2014, 2015, 2017; Astello‐García et al., 2015; De Santiago et al., 2018; Msaddak et al., 2017; Rocchetti et al., 2018). Young cladodes have a higher content of polyphenols than mature ones (Figueroa‐Pérez et al., 2018).

### 4.1 Extraction techniques for the analysis and characterization of polyphenols

Due to the high fiber content of cladodes (Table 1), other minor compounds (of equal biological importance), such as polyphenols, have not been studied deeply. Therefore, we reviewed the methods to extract and characterize polyphenols. Types of extraction methods include liquid‐solid extraction (a procedure that consists of grinding, defatting, solvent extraction, centrifugation, filtration, evaporation, and drying) (Yang et al., 2018) methanol/water/acid, methanol/acetone/water, and methanol/formic acid‐based techniques and are optimized by varying methanol concentrations between 50% and 80% (Table 3). For instance, Antunes‐Ricardo et al. (2014, 2015, 2017) extracted polyphenols with 4 N NaOH (1:10, m/v) at 40℃. Another study carried out by column chromatography, showed that a combination of 45℃ and airflow allowed optimal preservation of phenols and flavonoids (Medina‐Torres et al., 2011). The cladodes extracted by ethanol exhibited good solubility in polar solvents because the polar compounds act as scavengers against reactive oxygen species (Bonilla Rivera et al., 2017). Lastly, the Soxhlet and maceration method conducted by Ammar et al. (2015) in which variability in the extracts yields was attributed to the different polarities of the solvents used; in particular, the methanol and water extract produced the highest extraction yields.

**TABLE 3:** Analytical methods for the determination of polyphenols in cladodes

*Opuntia* species	Extraction method	Analysis	Compounds identified	Reference
*Opuntia ficus‐indica (L.)* *Mill*.	Alkaline hydrolysis: 4 N NaOH (1:10, m/v) at 40℃ for 30 min	Detection method: LC/MS‐TOF. Stationary phase: Zorbax SB‐C18, 3.0 × 100 mm, 3.5 µm. Mobile phase: A: water—formic acid, B: methanol. Mode: [M]^+^; 100–1500 m/*z*	Quercetin glucosyl‐rhamnosyl‐pentoside, isorhamnetin dihexosyl‐ rhamnoside, kaempferol rhamnosyl‐rhamnosyl‐glucoside, isorhamnetin‐glucosyl‐rhamnosyl‐rhamnoside, isorhamnetin‐glucosyl‐rhamnosyl‐pentoside, isorhamentin hexosyl‐methyl pentosyl‐pentoside, isorhamentin glucosyl‐pentoside, kaempferol‐glucosyl‐rhamnoside, isorhamentin glucosyl‐rhamnoside	Antunes‐Ricardo et al. (2014)
*Opuntia ficus‐indica (L.)* *Mill*.	Solvent extraction: 0.1 g of sample in 2 ml of methanol:acetone:water (5:4:1), 2.5 hr at 4℃	Detection method: LC–MS/MS. Stationary phase: Hydro‐RP18, (150 mm × 4.6 mm × 3 mm). Mobile phase: A: acetonitrile/methanol‐formic acid, B: formic acid. Chromatograms recorded: *λ* = 200–600 nm	Eucomic acid, chlorogenic acid, chlorogenic acid derivative, quercetin 3‐*O*‐rhamnosyl‐ (1→2)‐[rhamnosyl‐(1→6)]‐glucoside, quercetin 3‐*O*‐xylosyl‐ rhamnosyl‐glucoside, quercetin 3‐*O*‐dirhamnoside, kaempferol 3‐*O*‐(rhamnosyl‐galactoside)−7‐*O*‐rhamnoside, kaempferol 3‐*O*‐(rhamnosyl‐glucoside)−7‐*O*‐rhamnoside, kaempferol 3‐*O*‐robinobioside‐ 7‐*O*‐arabinofuranoside, isorhamnetin 3‐*O*‐rhamnoside‐ 7‐*O*‐(rhamnosyl‐hexoside), quercetin 3‐*O*‐rutinoside, quercetin 3*‐O*‐glucoside, isorhamnetin 3‐*O*‐rutinoside, kaempferol 3‐*O*‐rutinoside, quercetin 3‐*O*‐ arabinofuranoside, kaempferol 3‐*O*‐glucoside, kaempferol 7‐*O*‐neohesperidoside, isorhamnetin 3‐*O*‐galactoside	Astello‐García et al. (2015)
*Opuntia ficus‐indica (L.)* *Mill*.	Alkaline hydrolysis: 4 N NaOH (1:10, m/v) at 40℃ for 30 min	Detection method: HPLC‐PDA. Stationary phase: Zorbax SB‐C_18_ (9.4 × 250 mm, 5 µm). Mobile phase: A: water—formic acid, B: methanol. Mode: [M]^+^;100–1500 *m/z*	Isorhamnetin‐glucosyl‐pentoside, isorhamnetin‐glucosyl‐rhamnoside, isorhamnetin‐glucosyl‐rhamnosyl‐rhamnoside, isorhamnetin‐glucosyl‐rhamnosyl‐pentoside	Antunes‐Ricardo et al. (2015)
*Opuntia ficus‐indica* f. inermis	Maceration: 25 g of sample, ethanol 100%, 24 hr	Detection method: LC‐HRESIMS. Stationary phase: RP Pursuit XRs ULTRA 2.8, C_18_, 100 mm ×2 mm. Mobile phase: A: formic acid‐water, B: formic acid‐metanol. Mode: [M]^+^ 100–2,000 *m/z*	Quercetin, quercetin 3‐O‐glucoside, kaempferol, kaempferol 3‐O‐glucoside, kaempferol 3‐O‐rutinoside, isorhamnetin, isorhamnetin 3‐O‐glucoside, isorhamnetin 3‐O‐neohesperidoside, 3,3ʹ,4ʹ,5,7‐pentahydroxy‐flavanone, p‐coumaric acid, zataroside‐A, indicaxanthin, ß‐sitosterol	Msaddak et al. (2017)
*Opuntia ficus‐indica (L.)* *Mill*.	Solvent extraction: 4 g of sample, methanol 50%, 2 hr	Detection method: HPLC‐DAD. Stationary phase: Kinetex C_18,_ 5 µm RP 250 × 4.60 mm. Mobile phase: A: water—formic acid, B: acetonitrile. Chromatograms recorded: Phenolic acids: *λ* = 256–325 nm, flavonoids: *λ* = 360 nm	Quercetin, kaempferol, isorhamnetin, ferulic acid, 4‐hydroxybenzoic acid	De Santiago et al. (2018)
*Opuntia ficus‐indica*	Agitation: 4 g of sample, 0.1% formic acid in 80:20 (v/v) methanol/water, 25,000 rpm for 3 min	Detection method: UHPLC‐ESI‐QTOF‐MS; Stationary phase: Agilent Zorbax eclipse plus C_18_, 50 × 2.1 mm, 1.8 µm. Mobile phase: A: water, B: methanol‐formic acid‐ammonium formate. Mode:[M]^+^ 50–1000 *m/z*	Luteolin‐glu, apigenin‐glu, isoflavonoid, myricetin‐glu, quercetin‐glu, kaempferol‐Glu, isorhamnetin‐Glu, furofurans, dibenzylbutyrolactone, alkylphenols, hydroxybenzaldehydes hydroxycoumarins tyrosols, hydroxybenzoics, hydroxyphenylpropanoics, hydroxycinnamics	Rocchetti et al. (2018)
*Opuntia ficus‐indica (L.)* *Mill*.	Solvent extraction: 200 mg of sample, methanol acidified with formic acid, Sonicated for 25 min	Detection method: UHPLC‐ESI‐MS^n^. Stationary phase: XSelect HSS T3, (50 × 2.1 mm × 2.5 µm). Mobile phase: A: acetonitrile—formic acid, B: acidified acetonitrile. Mode: [M]^−^: noncolored phenolics, [M]^+^: Betalains	Protocatechuic acid hexoside, myricetin‐hexoside, ferulic acid derivative, ferulic acid hexoside, sinapic acid hexoside, quercetin‐rhamnose‐hexoside‐rhamnose, rutin‐pentoside, syrinigyl(t8‐*O*−4)guaiacyl, kaempferol‐di‐rhamnose‐hexoside, isorhamnetin‐ rhamnose‐rutinoside, quercetin‐hexoside‐pentoside, isorhamnetin derivative, dihydrosinapic acid hexoside, quercetin 3‐*O*‐rutinoside (rutin), secoisolariciresinol‐hexoside, isorhamnetin derivative, quercetin‐hexoside, kaempferol‐rutinoside, syringaresinol, naringenin‐hexoside, isorhamnetin rutinoside, isorhamnetin‐*C*‐hexoside, naringin	Mena et al. (2018)
*Opuntia ficus‐indica (L.)* *Mill*.	Maceration: 500 mg in 25 ml aqueous methanol (80%) overnight at 4℃	Detection method: LC/MS‐TOF. Stationary phase: Agilent Extended C_18_ (1.8 μm, 2.1 × 50 mm). Mobile phase: A: water +0.1% formic acid, B: acetonitrile. Mode: [M]^−^: 50–1,700 *m/z*	Piscidic acid, eucomic acid, isorhamnetin rhamnosyl‐rutinoside, isorhamnetin‐glucosyl‐rhamnosyl‐pentoside, rutin, narcissin (isorhamnetin rutinoside), isorhamnetin glucoside	Blando et al. (2019)
*Opuntia ficus‐indica*	Solvent extraction: 100% methanol (3× 2L)	Detection method: HPLC‐PDA‐Ms/Ms; Stationary phase: HS F5 column (15 cm 4.6 mm ID, 5 mm; Mobile phase: A:Water +0.1% formic, B: Acetonitrile +0.1% formic; Mode:[M]^+^	Quinic acid, malic acid, piscidic acid, diferuloyl‐syringsic acid, eucomic acid, dicaffeoylferulic acid, *p*‐coumaric acid 3‐*O*‐glucoside, 7‐glucosyl‐oxy−5‐methyl flavone glucoside sinapic acid 3‐*O*‐glucoside, sinapic acid 3‐*O*‐galactoside, quercetin pentosyl‐rutinoside, kaempferol rhamnosyl‐rutinoside isorhamnetin‐glucosyl‐rutinoside, rhamnetin rhamnosyl‐rutinoside, isorhamnetin rhamnosyl‐rutinoside, isorhamnetin pentosyl‐rutinoside, rutin, kaempferol pentosyl‐rutinoside, isorhamnetin pentosyl‐rutinoside, isorhamnetin pentosyl‐hexoside, isorhamnetin rutinoside, rhamnetin 3‐*O*‐glucoside, isorhamnetin 3‐*O*‐glucoside, isorhamnetin coumaroyl‐rutinoside, rhamnetin, isorhamnetin, diosmetin, tricin, hydroxyl octadecadienoic acid, eicosanoic acid, eicosanoic acid isomer heneicosanoic acid eicosanoic acid isomer behenic acid	El‐Hawary et al. (2020)

Note

HPLC‐PDA: high‐pressure liquid chromatograph equipped with a photodiode array detector; UHPLC‐ESI‐QTOF‐MS: ultrahigh‐performance liquid chromatography coupled with electrospray ionization‐quadrupole time‐of‐flight mass spectrometry; HPLC‐PDA‐Ms/Ms: High‐performance liquid chromatography‐photodiode array‐electrospray ionization mass spectrometry; LC/ MS‐TOF: liquid chromatography coupled to a time‐of‐flight mass spectrometer; UHPLC‐ESI‐MS^n^: ultrahigh‐performance liquid chromatography with electrospray ionization tandem mass spectrometry; LC–MS/MS: high‐performance liquid chromatographic and mass spectrometric; HPLC‐DAD: high‐performance liquid chromatography‐diode array detector system; LC‐HRESIMS: liquid chromatography‐high resolution electro‐spray ionization mass spectrometry.

Obtaining polyphenol‐rich extracts requires sample purification by column chromatography (Nemitz et al., 2015). Chromatography is a physical separation method based on differential migration of the sample components carried by the mobile phase through a stationary phase arranged in a column (Granato et al., 2016). Four types of chromatography can be applied to determine the polyphenolic profile of crude extracts from biological materials: high‐performance liquid chromatography (HPLC), thin‐layer chromatography (TLC), high‐performance thin‐layer chromatography (HPTLC), and capillary electrophoresis (CE) (Agatonovic‐Kustrin et al., 2020; Gadioli et al., 2018).

The highest extraction yields were obtained when using C18 reversed‐phase HPLC columns (with inner diameter 2–250 mm; particle size 1.8–2 5 μm) and a mobile phase composed of methanol or acetonitrile under isocratic elution or gradient elution (i.e., water and 0.1%–10% acetic or formic acid) conditions (Table 3). However, a factor to consider is the production of raw extracts, in which the management of parameters such as extraction time, temperature, and solvent composition influence the concentration and types of compounds obtained.

### 4.2 Identification of polyphenols

Several authors have identified polyphenols in cladodes through HPLC and UHPLC because they maximize polyphenol identification accuracy (Tan & Fanaras, 2018). Hence, these techniques lead the separation methods for polyphenols analysis (Table 3).

A study conducted by Petruk et al. (2017) with extracts from *Opuntia ficus‐indica* var. *saboten* found three phenolic acid derivatives: piscidic, eucomic, and 2‐hydroxy‐4‐(4‐hydroxyphenyl)‐butanoic acid. Astello‐García et al. (2015) identified polyphenols via LC‐MS according to retention time, UV spectra, and mass (*m*/*z*). Through the aglycone fragment, they examined the structure of each flavonoid by characterizing quercetin ([M]^+^ *m*/*z* 301), isorhamnetin ([M]^+^ *m*/*z* 315), kaempferol ([M]^+^ *m*/*z* 285), and luteolin ([M]^+^ *m*/*z* 285).

Similarly, Antunes‐Ricardo et al. (2015) found isorhamnetin glycosides by HPLC‐PDA. Rocchetti et al. (2018) detected 89 flavonoids—mostly the glycosidic forms of kaempferol, isorhamnetin, and quercetin—and 54 phenolic acids in cladodes. This was the first evaluation that includes the phenolic profile in cladodes using UHPLC‐ESI/QTOF‐MS. Msaddak et al. (2017) studied an ethanolic extract of cladodes utilizing LC‐HR‐ESI‐MS; they found 9 flavonoids and 2 phenolic acids. Furthermore, Mena et al. (2018) identified flavanones and lignans by UHPLC‐ESI‐MS^n^ and observed a higher polyphenol content in young cladodes compared with matures ones, which could be attributed to physiological modifications.

Spectrometry‐based techniques are a powerful and fast tool to accurately differentiate compounds in food matrices. However, it is unreliable when quantifying polyphenols due to the lack of availability of reference standards. Among the analytical methods compared in this review, UHPLC‐ESI/QTOF‐MS exhibited an outstanding performance to identify the main polyphenolic classes and subclasses in cladode extracts. Simultaneously, it detected multiple compounds based on the mass‐to‐charge ratio (*m/z*) of a molecular ion ([M − H]‐) and the characteristic production for each polyphenol.

## 5 CONCLUSIONS AND FUTURE PERSPECTIVES

The present study reviewed the structure function of cladodes, which may provide an nutritional and functional value given the properties of their major chemical components. Several studies have shown that polyphenols in cladodes are associated with beneficial effects on human health. Polyphenols can be separated and identified by conducting advanced analytical techniques, which have different advantages associated with the solute‐solvent ratio. Here, we described diverse processes in current research to detect polyphenols in cladodes that could be implemented in future technological developments. The forthcoming research should focus on obtaining additional information to standardize the analytical methods designed to categorize and quantify the polyphenols in cladodes; and conducting more experimental studies, such as in vivo models, on polyphenol cladode extracts to determine the characterization of nopal biological activity.

## CONFLICT OF INTEREST

The authors declare that there is no conflict of interest.

## AUTHOR CONTRIBUTIONS

Madeleine Perucini‐Avendaño: Conceptualization (lead); Investigation (lead); Writing‐original draft (lead). Mayra Nicolás‐García: Conceptualization (supporting); Investigation (supporting). Cristian Jimenez: Conceptualization (equal); Investigation (equal); Supervision (lead); Writing‐review & editing (supporting). Maria de Jesús Perea‐Flores: Formal analysis (supporting); Supervision (supporting). Mayra Beatriz Gómez‐Patiño: Visualization (supporting). Daniel Arrieta‐Baez: Visualization (supporting). Gloria Davila‐Ortiz: Project administration (lead); Supervision (supporting); Writing‐review & editing (lead).

## ETHICS APPROVAL

Studies involving animal or human subjects were not required for this review.
